# C-myc and N-myc in the developing brain

**Published:** 2010-05-29

**Authors:** Alice Wey, Paul S. Knoepfler

**Affiliations:** Institute of Pediatric Regenerative Medicine, University of California Davis School of Medicine, Department of Cell Biology and Human Anatomy, Shriners Hospital For Children Northern California, Sacramento, CA 95817

**Keywords:** Wey and Knoepfler. c-myc and N-myc promote active stem cell metabolism and cycling as architects of the developing brain. OncoTarget 2010. 1

**Figure 1.**
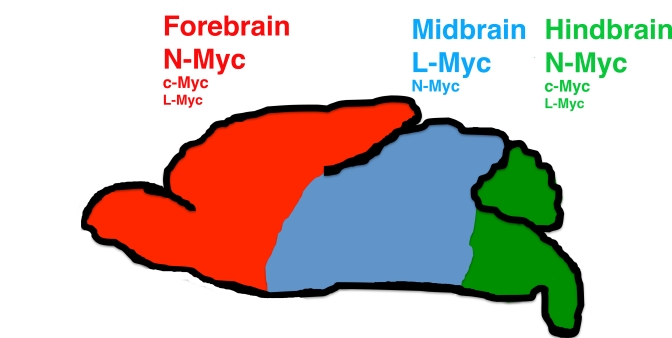
Model of regulation of brain architecture by the myc gene family.

Murine knockouts (KO) of myc genes have provided substantial insight into not only their normal functions during development, but also shed light on their tumorigenic functions when they are misregulated.  c-myc and N-myc genes both are essential in neural stem and precursor cells (NSC) for normal mouse brain growth[1,2]. The fact that mutation of N-myc also causes microcephaly in humans in Feingold Syndrome[3], suggests common Myc-regulated pathways are at work during mouse and human brain growth. Earlier studies indicate that N-Myc drives brain growth by maintaining widespread domains of euchromatin [4] required for NSC proliferation and pluripotency. Such events may be "locked in" during tumorigenesis driving Myc-associated neuronal tumors such as neuroblastoma and medulloblastoma as N-Myc regulates global euchromatin in neuroblastoma as well [5]. A substantial obstacle to understanding the respective functions of different myc genes in neurogenesis is their redundancy and coexpression. To overcome these problems and explore Myc function in brain development as well as tumorigenesis, here we generated a double KO (DKO) for c- and N-myc using nestin-cre. Overall brain growth is strongly impaired in DKO mice, a phenotype associated with decreased cell cycling and migration of NSC, which are profoundly decreased in number. Interestingly, the midbrain of DKO mice appeared unaffected by loss of c- and N-myc, suggesting that L-myc controls midbrain growth, while both c- and N-myc regulate fore- and hindbrain development (Figure 1). Overall, N-myc is the main conductor of brain growth. Surprisingly, survival was not clearly affected by the loss of c- and N-myc in NSC. Changes in gene expression in the DKO include downregulation of genes involved in protein and nucleotide metabolism, mitosis, and chromatin structure as well as upregulation of genes associated with differentiation. Very few changes in gene expression were observed in the midbrain, further supporting the notion that c- and N-myc are dispensable for midbrain development. These are the first in vivo studies of Myc regulation of gene expression in the nervous system. Our findings suggest a model of neuronal tumorigenesis in which excess myc aberrantly enforces a developmentally active chromatin state characterized by rapid cell cycling, hyperactive metabolism, and blocked differentiation. We refer the readers to the full text paper [6].
